# The Operation for Adenoids

**Published:** 1908-04-11

**Authors:** 


### Laryngology and Rhinology.

### THE OPERATION FOR ADENOIDS.

The removal of adenoids is generally considered to be a very simple and easy operation. It is certainly easy, but it becomes so only by careful attention to every detail, and after considerable practice. The many different positions and methods of procedure which have been recommended are sufficient proof that the operation is not one of absolute simplicity. Indeed, everyone who has had to teach the performance of this operation to students, and who has had the opportunity of seeing the after-results of a number of cases in a large out-patient department, will agree that to remove adenoid vegetations with thoroughness and certainty is not always a perfectly simple matter. Considerations of space will not permit a discussion of all the methods which have been, or still are, employed; but it may be useful to describe the mode of procedure which is coming more and more into general use among specialists, and to give a few hints about those details which are of value in actual practice.

The risk of the entry of blood, or even of portions of the growth, into the lungs is a very real one, and should ever be present in the mind of the surgeon about to perform any operation, however trivial, on the upper air-passages under anæsthesia. Even if the patient be saved from immediate suffocation after this accident, pneumonia is very likely to ensue, and that of a very virulent and fatal form. This is no imaginary danger, but has been known to occur after the removal of a small part of the inferior turbinated body. It is, however, a danger which can easily be averted, partly by avoiding deep anæsthesia, but chiefly by the correct position of the patient. At one time it was customary to place the patient on the back with the head extended and hanging over the end of the table. This position has been abandoned by most operators, for congestion and hæmorrhage are thus increased; the adenoids lie in a kind of hollow above the anterior tubercle of the atlas, the prominence of which often interferes with the sweep of the curette, and hyperextension of the cervical spine increases this difficulty. It is better to allow the patient to lie straight upon a flat table and to make the first sweep with the curette in this position, for then it is easy to keep the middle line, to use sufficient force, and to avoid injury to neighbouring structures. Immediately after the first sweep has been made the patient is turned completely on to his right side; as he is now lying on his shoulder, without a pillow, the larynx is at a higher level than the naso-pharynx and blood cannot enter the air-passages.

The best anæsthetic is the chloroform-ether mixture for children, and chloroform for adults, and in all cases the anæsthetist should have a Junker's apparatus ready so as to be able to continue the administration if required. It is sometimes the practice to give gas in the sitting position to patients over twelve or fourteen, but this is not to be recommended. Gas is in general a bad anæsthetic for cases of nasal obstruction; it increases congestion and hæmorrhage, and, though the anæsthesia is long enough for the skilful removal of adenoids and tonsils, it is insufficient to deal with tags which may be left or to remove the posterior ends of the turbinals, which are so often enlarged in older patients who have adenoids: it is quite unsuitable for young children. If gas be used at all, it should be given in the recumbent position; the sitting posture allows the blood to run from the mouth if the head be bent slightly forward, but the rigidity which often follows gas, especially in cases of obstruction, adds considerably to the difficulties of the operation.

The old Gottstein curette has several defects which have been corrected in later modifications. The instrument should have a straight back, and only be curved to a little beyond a right angle, and, above all, its arms should be parallel and not Y-shaped, for in the latter form the vegetations tend to be pushed away from the knife, and the extremities of the cutting blade are not so completely under control, and may injure the Eustachian tubes. Several good patterns have a hinged cage attached to the blade; this contrivance has the great advantage of bringing out the mass of the adenoid entire, but it is not essential, for the growth can be brought out whole by an unguarded curette if the proper swing be used.

The patient is placed on the back, with the head quite straight, and the mouth opened widely with a gag. Doyen's pattern is the best if tonsils have also to be removed; it should be placed on the left side of the mouth, and its plates should be well to the left of the middle line, so as to allow the curette to be depressed on to the central incisor teeth. The operator stands at the patient's right side, and the nurse at his left. While the left forefinger retracts the soft palate, the curette is passed behind, held firmly like a dagger; the handle must be fully depressed, and the cutting-blade raised until it meets the back of the nasal septum, for the commonest error is to leave behind the upper and most obstructive part of the growth. The shaft of the curette is now raised against the upper incisors, while the blade sweeps down the naso-pharynx; it must not be dragged straight downward or tags will be left; but the cutting end should be made to describe the segment of a circle about the handle, so that the knife leaves the pharynx at an angle, cutting cleanly through the lower end of the growth and bringing it right out of the mouth. The patient is now turned on to the right side, and this must be done at once, whether the growth has been successfully removed or not. Adults who are heavy to move may be placed at the outset upon the right side; if the surgeon stands behind the head and holds the curette in the fist, with the thumb nearest the blade, he will find that he can made the requisite powerful circular sweep without much difficulty. The naso-pharynx can now be explored at leisure with the finger, and a cageless curette may be introduced if necessary to complete the removal. In patients over the age of ten or twelve a couple of snares should always be ready; the posterior ends of the turbinals should be examined, and removed if found enlarged; hypertrophy of these parts is very common in cases of adenoids of long standing, and is the chief cause of disappointment after the adenoid operation. The tonsils, if enlarged, should then be removed with a plain spade guillotine without barbs, while the patient is in the lateral position. It is most advisable to use a good light and a forehead mirror, or, better still, an electric head-lamp, and to examine the throat carefully before the patient becomes conscious, making sure that the tonsils are completely removed, and that no tags of adenoid remain. The latter may most readily be removed with punch-forceps. There is no need for hurry during this latter part of the operation; more anæsthetic may be given if required, and the patient is in a perfectly safe position. It is very unpleasant to have to remove a tag from a nervous frightened child after he has come round, more especially as the parents may believe and repeat that "the first operation was unsucessful, and had to be repeated."

